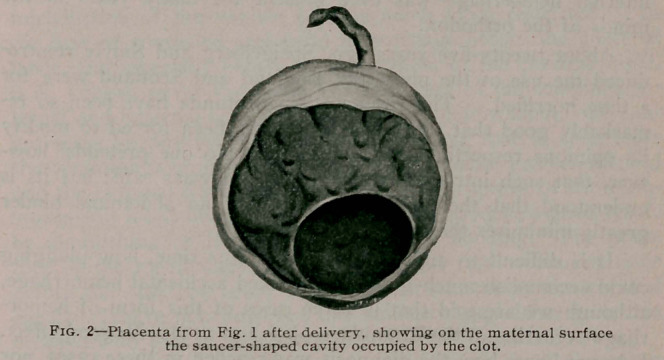# Some Points in the Diagnosis and Treatment of Accidental Hemorrhage1Read at the nineteenth annual meeting of the American Association of Obstetricians and Gynecologists, held at Cincinnati, September 20-22, 1906.

**Published:** 1906-12

**Authors:** Adam H. Wright

**Affiliations:** Professor of Obstetrics, University of Toronto, Canada


### Some Points in the Diagnosis and Treatment of Accidental Hemorrhage.[1]

BY ADAM H. WRIGHT, B.A., M.D., M.R.C.S., Eng.
Professor of Obstetrics, University of Toronto, Canada.

[*From American Journal of Obstetrics, November 1906.*]

ACCIDENTAL hemorrhage is one of the most complex subjects in obstetrics, and one of the causes of its complexity is the word hemorrhage. In medicine and surgery we consider certain hemorrhages, such, for instance, as one in the brain or pancreas, dangerous because of the resulting clots which act as foreign bodies, and by irritating pressure, produce serious effects. In obstetrics most practitioners consider that the serious condition in all forms of what is technically known as accidental hemorrhage, is loss of blood.

---

1. Read at the nineteenth annual meetlng of the American Association of Obstetricians and Gynecologists, held at Cincinnati, September 20-22, 1906.

My aim in this paper is to refer simply to certain points in connection with accidental hemorrhage, without any attempt to treat the subject in a complete or systematic manner. In a paper on the concealed variety read at the meeting of the British Medical Association last August, I reported a case of the concealed form. I may say that four cases of concealed accidental hemorrhage have come under my observation.

As these cases are exceedingly rare, I take the liberty of repeating one report of great interest, because I had the opportunity of watching the patient carefully from the onset of the serious symptoms until the time of her complete recovery.

Patient, aged 29, II-para, when seven months advanced in pregnancy was suddenly seized with abdominal pain while driving. Went home (only a short distance) as soon as possible. Went upstairs intending to go to bed, collapsed while undressing. I saw her in about twenty minutes and found her cold and weak, pale, with rapid pulse, and suffering intensely from "tearing" pains over the abdomen. Gave her large doses of morphine and ordinary treatment for shock. The symptoms, although very alarming for about two hours, subsided. Concealed hemorrhage suspected at first, but doubted on following day. Four days afterward an uneventful labor resulted in the expulsion of a dead fetus. The placenta showed evidence of being nearly half detached. The shock was evidently due to the tearing pains caused by the sudden impingement on the uterine wall, and not at all to the loss of blood, which did not amount to more than a pint (I think less than a pint). The clot thus produced, although comparatively small, had acted as a foreign body, and produced a surgical traumatism which nearly caused the death of the patient.

In considering the whole subject of accidental hemorrhage, several differences of opinion are prevalent, both as to diagnosis and treatment. The following procedures are conspicuous in this regard,—rupture of the membranes, plugging the vagina, accouchement forcé, and Cesarean section. In this paper vaginal Cesarean section will not be considered as a variety of accouchement forcé. Why is there so much divergence of opinion? In the first place, probably because the internal concealed variety is absolutely different from the external hemorrhage as to symptoms and results, and requires entirely different treatment.

Again, all accidental hemorrhages are at first internal and concealed, and present a great variety of symptoms, such multiplicity depending on the suddenness of the gush, the amount of blood poured out, and the length of time of concealment. The blood streaming from the ruptured vessels may flow freely from the uterus at once, or may be bottled up for a varying time.

In a certain proportion of cases of accidental hemorrhage there is little or no shock, because there is little or no obstruction to the flow of blood. The important condition here is loss of blood resulting in collapse, with no tearing or agonising pains.

What is shock? We have no time now to discuss such an important subject in detail, but we recognise the fact that the surgeons of today are giving us valuable instruction as to this condition. Obstetricians have said much about shock as produced by accidental hemorrhage during the last sixty years. Many of their statements have been rather vague, but they generally indicate that shock, in connection with accidental hemorrhage, is a condition produced by loss of blood. It will simplify the matter somewhat from the standpoint of this paper to leave out of the question what is known as post-operative shock. I shall, however, give a definite meaning to the two words, shock and collapse, although it will be admitted that any distinction between these two conditions must be to a certain extent artificial.

Shock will be considered a condition which is produced more or less suddenly by a serious traumatism, or surgical injury, similar, for instance, to that produced by the wheel of a railroad car passing over and crushing a limb.

Collapse will be considered a condition caused by extreme exhaustion, especially that induced by severe hemorrhage, and depending on the amount of blood lost. It is, of course, difficult in many cases to differentiate between these two conditions. It may be that we have shock from traumatism and collapse from hemorrhage to an equal degree in certain cases of concealed hemorrhage.

In the majority of cases of concealed hemorrhage, however, shock and not loss of blood is probably the prominent symptom or condition. The muscle of the uterine wall is generally strong enough to resist the impact of the *new body* without appreciably stretching, while the nerves suffer from the sudden pressure to such an extent that a severe storm arises, causing agonising pain, intense shock, and tetanic spasm of the uterine musculature, with a cervix "as hard as iron."

There is another entirely different class of cases where the uterine wall is not strong enough to offer much resistance, and as a consequence the uterine cavity enlarges greatly, allowing an escape of blood from the uterine vessels sufficient to destroy life. I have not seen, nor have I met a physician who has seen such distention of the uterus, but we must accept the evidence of such careful observers as Oldham of Guy's Hospital, and others who have recorded such cases.

It should not be difficult to recognise such a condition because the patient would steadily grow worse, the abdomen would become enlarged, the temperature would be lowered, but the pulse rate would grow more rapid.

Diagnosis.—No attempt will be made to deal fully with the important subject of diagnosis. We may consider that, in many cases of the concealed, combined internal and external, and the ordinary external varieties, diagnosis is often difficult or impossible for a time at least. The diagnosis of the concealed form is often impossible until after delivery.

In my paper of last August I referred to a very admirable discussion on accidental hemorrhage at the meeting of the British Medical Association two years ago. I can hardly agree, however, with the opinions there expressed respecting the diagnosis of the concealed form.

Sir Arthur McCann expressed the opinion that in many cases the diagnosis is impossible until after the expulsion of the placenta; but he adds: "However, once the symptoms of anemia are well marked, and are accompanied with much pain and tension or localised swelling, there is usually not much trouble about the diagnosis."

Jellett, who expresses the latest views from Dublin, tells us the symptoms of concealed hemorrhage fall under two heads: (1) "Those due to loss of blood;" (2) "those due to the accumulation of blood in the uterus." He adds that "the most prominent symptom in the second grade consists in the gradual enlargement of the uterus." Similar views are generally entertained in the United States and Canada. Whitridge Williams tells us,—"The appearance of acute anemia with manifestations of shock in a patient in the later months of pregnancy should always suggest the concealed uterine hemorrhage."

While it must be admitted that acute anemia and uterine distention are sometimes present, it seems certain that in a case such as I have described, there is no acute anemia or marked distention of the uterus. Therefore, these two symptoms to which so much prominence is given by many, if not by most authors, should receive less consideration in such cases.

What, then, are the symptoms of concealed accidental hemorrhage? They are probably in the majority of cases pain and shock. Pain generally comes on suddenly,—so suddenly sometimes that it resembles the "solar-plexus punch" that "knocks out" the prize fighter. The pain is so entirely different from the ordinary labor pain that the patient and her friends generally realise that something serious has happened. The pain is continuous instead of being intermittent, amounts to extreme agony and is accompanied by tetanic contraction of the uterine walls, including both body and neck. These symptoms should lead us to suspect that concealed hemorrhage is the possible or probable cause of the patient's condition.

Of course, one may be mistaken, as the following case will show: Mrs. A., aged 25, II-para. In the eighth month of pregnancy was suddenly seized with extreme continuous pain, accompanied by tetanic uterine contraction, July 24, of this year. When I saw the patient I suspected concealed accidental hemorrhage. Morphine was injected and the woman was at once sent to a private hospital, where she received a second dose of morphine and suitable treatment for shock. On the following day there was some local tenderness over the abdomen, with slight elevation of temperature, and catarrhal appendicitis was then suspected. In addition to the administration of morphine, the patient received calomel and castor oil, and recovered in about eight days (no signs of labor appearing in the meantime), and was sent home. On the last day of August labor commenced. She returned to the private hospital and, after a somewhat tedious labor, a healthy child was born September 1. The patient made a good recovery. Careful examination of the placenta revealed no trace of "old clot."

Althought one may make a mistake in diagnosis in such cases, the prompt and appropriate treatment of pain and shock, or in other words, the treatment of symptoms according to the methods of many able obstetricians of fifty or sixty years ago appears to be correct.

Treatment.—In the paper on the concealed variety, reference was made to Goodell's extremely valuable and interesting paper on the same subject. This distinguished obstetrician described in a very graphic way the symptoms, but he did not, in my opinion, properly differentiate between shock from traumatism and collapse from loss of blood; and as a consequence, his advice in such cases, "to deliver the woman as soon as possible," impelled men to carry out very radical forms of delivery with disastrous results.

The following is a synopsis of the treatment I have recommended when traumatic shock is the chief factor.

1. Administer morphine by hypodermic injection, half a grain at once, a quarter of a grain in half an hour after, and another quarter after another half hour, or less, if required; that is, one grain within an hour. Atropine may be given as well, if thought advisable.

2. Lower the patient's head and elevate the foot of the bed.

3. Keep up the body temperature by the external application of artificial heat.

4. Give a high enema of salt solution. Subcutaneous or intravenous injections may sometimes be advisable.

5. Give small doses of strychnine (not more than two doses, of one-thirtieth of a grain each, by hypodermic injection).

The latter recommendation is given with some confidence, notwithstanding the adverse views of certain surgeons. I

FIG. 1.—Pregnant uterus, seven months, membranes intact, uterine wall tetanically contracted, cervix "hard as iron," showing a post-placental clot between a portion of the placenta and the uterine wall.

believe at the same time that large doses of this medicine are exceedingly dangerous in certain cases of either shock or collapse.

In the discussion which followed the reading of my paper at the Toronto meeting, it was stated, "that the same results could be obtained in many different ways. In Dublin the practice was to plug the vagina; in Edinburgh to rupture the membranes."

I had no opportunity to reply, but I desire now to call attention to the fact that in one of the cases reported (which is also reported today), the clot resulting from the outpour of blood was entirely post placental, the whole margin of the placenta being adherent to the uterine wall. (Fig. 1). Neither the vaginal plug nor the puncture of the membranes could in this case mitigate the serious symptoms of pain and shock.

The combined internal and external accidental hemorrhage, whether occurring before or during labor, is more easily diagnosticated than the purely concealed form; but in certain cases, the diagnosis is sufficiently difficult to cause much perplexity. When, for instance, a quart of blood is retained internally and only two ounces escape externally, we have a condition closely similar to the concealed variety, with intense pain and shock. Under such circumstances it seems reasonable to suppose that the pain and shock are still the prominent symptoms, and should receive very prompt treatment. After complete or partial recovery from pain and shock it would seem well to carry out

FIG. 2—Placenta from Fig. 1 after delivery, showing on the maternal surface the saucer-shaped cavity occupied by the clot.

the so-called Dublin treatment to which further reference will be made, or perform a vaginal Cesarean section. A report of a case with similar conditions and symptoms will be given later.

In what is known as the external accidental hemorrhage, there is little or no shock, but there may be collapse from loss of blood. It is probably in such cases that the greatest difference of opinion prevails, especially as to two procedures, rupture of the membranes, and the introduction of the vaginal tampon. The puncture of the membranes and the use of the plug are very old procedures in the treatment of accidental hemorrhage.

We are told that one hundred and thirty years ago there was a great difference of opinion on this question, and at that time Leroux, of Dijon, was a strong advocate of "rupture of the membranes," and the "administration of ergot of rye to check the flooding."

The discussions as to these two methods during the first and second thirds of the last century were interesting, and were equal in many respects, and perhaps better in some respects, than the discussions of the last forty years. It would appear from the discussion last August in Toronto that we have not made much progress in a century and a half as to the proper estimate of these two procedures. There can be little or no doubt, however, that each method is good in its proper place.

All things considered, the vaginal plug appears to be more generally useful, and safer, for the various kinds of accidental hemorrhage than any other procedure. And yet, a candidate at a final examination, who expressed such an opinion before a board of examiners in London, England, ten years ago, would probably have been plucked because of such dangerous heterodoxy. That terrible danger of converting an external into an internal hemorrhage was ever present for many years in the minds of the orthodox.

About twenty-five years ago, Speigelberg and Smyly reintroduced the use of the plug, and England and Scotland were for a time horrified. The results at the Rotunda have been so remarkably good that the whole world has been forced to modify its opinions respecting the procedure. No one pretends, however, that such introduction of a plug is always safe; but its is understood that the proper application of an abdominal binder greatly minimises the dangers.

It is difficult to understand, at the same time, how plugging could accomplish much good in concealed accidental hemorrhage, although we are told that in three cases of this form of hemorrhage occurring at the Rotunda, a plug was used with good effect. I do not know how the diagnosis was reached in these cases, nor when the plug was introduced; but I should suppose that such introduction would be absurd while the patient was suffering, perhaps dying, from shock.

If I should again meet a case similar to that which I have reported in which severe pains came on four days before the onset of labor, I should now be inclined to interfere after the patient had rallied, say on the day after the advent of the acute symptoms. After such a severe nerve storm as I have described, with its prolonged condition of uterine tetanic spasm, a fetus is, so far as I know, always dead.

Although I believe that the statement made by Goodell, that in such cases the rule should be imperative "to deliver the woman as soon as possible," has done a large amount of harm, by encouraging the operation of accouchment forcé, even while the cervix is "as hard as iron," yet I believe that the patient can hardly be considered safe until the uterus is emptied. I think, therefore, when all symptoms of shock have disappeared, it might be well to introduce a bougie into the uterus, according to the Krause method (being careful not to rupture the membranes), and at the same time introduce a plug into the vagina. This can be done best by using some form of Sim's speculum, introducing a gum elastic or rubber bougie, about 12, English size, as far as possible, and packing the vault of the vagina with antiseptic gauze. I prefer 5 per cent. iodoform gauze.

It seems remarkable that rupture of the membranes in cases of accidental hemorrhage should have had so many advocates during the last one hundred and fifty years. While one of the oldest, it is one of the crudest, and one of the most objectionable obstetrical operations when performed before the onset of labor. There is no doubt, however, that the procedure is an excellent one in its proper place, but its scope as compared with that of the introduction of the vaginal plug is much more restricted. In the case of concealed accidental hemorrhage before the onset of labor, and especially before the effacement of the cervical canal, it is, I think, never justifiable. In the case of the combined internal and external accidental hemorrhage before labor, one cannot speak so positively, but it seems probable that in such cases the operation is unjustifiable. If, however, labor has commenced and the external hemorrhage is serious, the rupture of the membranes will frequently, or perhaps generally, produce a good result by diminishing or frequently stopping the flooding. The puncture of the membranes may produce good results even if done before the onset of labor if there is effacement of the cervical canal. I have not, however, had sufficient experience with this procedure to give a definite opinion in that regard.

It would not be profitable to spend much time discussing accouchement forcé. Its mortality rate, when the patient is suffering from shock, and the uterus is tetanically contracted, is, in my opinion, exactly 100 per cent. I know of no operation in obstetrics or surgery respecting which one can speak more definitely. A certain amount of force may, however, be used when the os is dilatable. For instance, we may have ruptured the membranes before the os is dilated; there may still be considerable hemorrhage, making an early emptying of the uterus desirable. In such a cases manual dilatation of the os, with version or forceps delivery, may be advisable. It is doubtful, however, whether the manual dilatation, under such circumstances, should be designated accouchement forcé.

I should like to discuss the operation of vaginal or abdominal Cesarean section, but I have had no experience in connection with either operation for accidental hemorrhage. In the case of either concealed, or combined internal and external accidental hemorrhage, with the patient suffering from shock and a cervix as "hard as iron," any operation would probably be unjustifiable. The shock should be properly treated; and if, in spite of such treatment, the symptoms of shock grow worse instead of better, the patient is probably going to die. If, on the other hand, the patient recovers from the shock, it is certain that in some cases, if not in the majority, serious operation is not advisable. It might happen, however, that internal hemorrhage was taking place into a uterus whose walls were stretching rapidly, and the loss of blood was the chief factor. If, in such an emergency, it was not possible to dilate the os, and deliver soon enough to save life, some form of Cesarean section might be deemed advisable. Vaginal section, in one of its varieties, seems the best radical operation in sight; but I am inclined to think its field is exceedingly limited. It seems to me entirely unsuited for such a case as I have reported today, especially when there is doubt as to the diagnosis; but it may be indicated in cases where labor is imminent, or present, especially where loss of blood, whether internally or externally, or both, is the serious factor.

I shall now submit to the Fellows of the Association a case reported something like ninety years ago: "A lady of weakly constitution and delicate habit, was attacked in the later months of pregnancy with a slight discharge of blood from the vagina, not amounting altogether to half an ounce, accompanied with alarming symptoms of exhaustion and debility. The os uteri was scarcely dilated to the size of a sixpence, and was in such a state of rigidity as precluded the possibility of affording any manual assistance. The lady in consequence died, and, on examination after death, it was found that the separation of the center of the placenta from the parietes of the uterus had taken place, whilst its edges were completely adherent, forming a kind of cul-de-sac into which blood had been poured, to the amount of a pint and a half, which had become coagulated within the cavity thus formed."

What would you do in such a case as this? Did this woman die from shock, or from loss of blood, or from a combination of the two? I have never met a case where the loss of a pint and a half of blood without other complication caused death; but I must admit that such a hemorrhage is a serious matter. I think, however, that the rigidity of the os was due to that peculiar nerve storm which is present in such cases, and that the patient died from shock. Under such circumstances, my chief aim would be to first treat the shock, as indicated elsewhere in this paper. As to operative procedures I should like to get your opinions.

The main points of this paper are as follows:

1. Making a diagnosis in many cases of concealed accidental hemorrhage is generally difficult, sometimes impossible, before delivery.

2. The so-called important symptoms,—anemia and distention of the uterus,—are not present in a large proportion of such cases.

3. The serious condition in most cases is shock from traumatism, and not collapse from loss of blood.

4. The diagnosis of the combined internal and external accidental hemorrhage is more readily made, but the amount and effect of the blood within the uterus are often difficult to ascertain.

5. Even in such cases, shock from traumatism is sometimes the predominating element; on the other hand, collapse from loss of blood, whether retained within the uterus or flowing externally, is sometimes the important factor.

6. In all cases where shock from traumatism is the main condition, or the predominating element, the most urgent requirement is proper treatment of such shock, and not emptying the uterus.

7. In a large proportion of cases of the combined internal and external hemorrhage, the introduction of the vaginal plug, with the application of an abdominal binder, appears to be a very safe and effectual plan of treatment.

8. In a small proportion of cases, especially during labor, puncture of the membranes is beneficial.

9. Any form of accouchement forcé, which includes forcible dilatation of a rigid cervix is never justifiable.

10. The best operative procedure would appear to be some form of vaginal section; but its field is limited, and not accurately defined.

30 Gerrard Street East.

---

Impotence caused by bromo-seltzer.—W. J. Robinson, New York (*Journal A. M. A.*, August 18), reports that a married man consulted him on account of loss of sexual power, but who also suffered from cardiac symptoms, digestive disorder, etc., and had severe headaches, for over a year. Inquiry developed the fact that he had been taking bromo-seltzer for a long time and was using an average two of the dollar-size bottles a week. The drug was voluntarily discontinued by the patient when informed as to its connection with his condition. The special action of bromo-seltzer on the sexual organs as seen in this case, he thinks has not been hitherto reported.

## Figures and Tables

**Fig. 1. f1:**
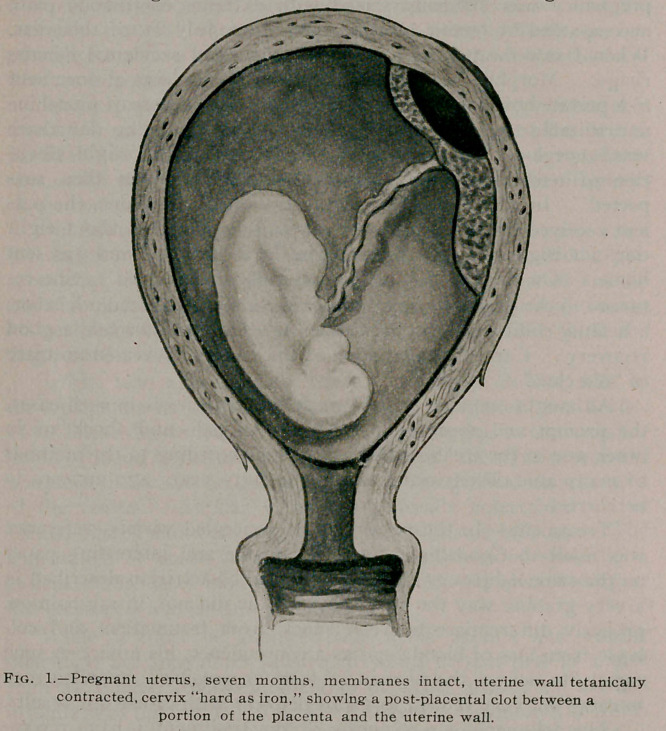


**Fig. 2. f2:**